# Dietary Acrylamide Exposure and Cancer Risk: A Systematic Approach to Human Epidemiological Studies

**DOI:** 10.3390/foods12020346

**Published:** 2023-01-11

**Authors:** Burhan Başaran, Burcu Çuvalcı, Güzin Kaban

**Affiliations:** 1 Department of Plant and Animal Production/Tea Agriculture and Processing Technology, Pazar Vocational School, Recep Tayyip Erdoğan University, Rize 53100, Turkey; 2 Health and Care Services/Elderly Care, Health Services Vocational High School, Recep Tayyip Erdoğan University, Rize 53100, Turkey; 3 Department of Food Engineering, Faculty of Agriculture, Atatürk University, Erzurum 25240, Turkey

**Keywords:** acrylamide, cancer, dietary exposure, human cancer

## Abstract

Acrylamide, identified by the International Cancer Research Center as a possible carcinogenic compound to humans, is a contaminant formed as a result of the thermal process in many foods, such as coffee, French fries, biscuits and bread, which are frequently consumed by individuals in their daily lives. The biggest concern about acrylamide is that the health risks have not yet been fully elucidated. For this reason, many studies have been carried out on acrylamide in the food, nutrition and health equation. This study focused on epidemiological studies examining the associations between dietary acrylamide exposure and cancer risk. For this purpose, articles published in PubMed, Isı Web of Knowledge, Scopus and Science Direct databases between January 2002 and April 2022 were systematically examined using various keywords, and a total of 63 articles were included in the study. Although some studies on reproductive, urinary, gastrointestinal, respiratory and other systems and organs stated that there is a positive relationship between dietary acrylamide exposure and cancer risk, many publications did not disclose a relationship in this direction. Studies examining the relationship between dietary acrylamide exposure and cancer should be planned to include more people and foods in order to obtain more reliable results. Making research plans in this way is very important in terms of guiding health policies to be formed in the future.

## 1. Introduction

Acrylamide is a compound formulated with an unsaturated carbonyl group C_3_H_5_ON, which is easily soluble in acetone, ethanol, methanol and water, has a low molecular weight (71.08 g/mol), and has a colorless, odorless, crystalline structure [1]. Since the 1950s, acrylamide derivatives have been widely used in dams, tunnels and underground construction works [2], and in other fields of industry such as water treatment, paper, textile, and as laboratory reagents [3]. The presence of acrylamide in food was revealed in Sweden in 2002 as a result of a series of studies carried out after a leak in a tunnel construction [4]. This discovery aroused great curiosity in the scientific world and much research was carried out in this field. These studies can be grouped under five main headings: acrylamide formation mechanisms in foods; acrylamide levels in foods; acrylamide reduction in foods; dietary acrylamide exposure; and the effect of acrylamide on health.

1. Acrylamide formation mechanisms in foods: Uncertainties regarding acrylamide formation mechanisms in foods still continue [5]. It is reported that acrylamide is formed at high levels, especially in foods rich in carbohydrates, through thermal process above 120 °C [6]. Although it varies according to the type of food, the Maillard Reaction is accepted as the primary mechanism in the formation of acrylamide [7]. Apart from this mechanism, other mechanisms are also described including glycerol, fatty acids, aspartic acid carnosine, B-alanine, pyruvic acid, serine and cysteine compounds, also called acrolein pathway, and resulting in acrylamide formation [8,9,10].
2. Acrylamide levels in foods: The level of acrylamide in foods varies depending on many factors such as the type and content of food, processing technique, and storage conditions [11]. In this context, much research, including traditional foods specific to societies, especially foods that are widely consumed around the world, has been carried out. In recent studies, acrylamide levels were found to be 779–1299 μg/kg in French fries [12], and 211–3515 μg/kg [13] in potato chips, which is quite high compared to the acrylamide levels in other foods. Acrylamide levels have been reported to be 31–454 μg/kg [14] in bread, 135–1139 μg/kg [13] in coffee, and 5.30–79.5 μg/kg in ready-to-drink (brewed) coffee [15]. It was determined to be <20–639 μg/kg [16], 15–109 μg/kg [17], and 12.3–1270 μg/kg [18] in breakfast cereal, infant formulas and baby biscuits, respectively. Acrylamide levels were reported to be <LOQ–143 µg/kg for some traditional Polish foods [19], and 11.7–527 μg/kg for some Turkish traditional foods [20].
3. Acrylamide reduction in foods: The detection of acrylamide in foods led to studies on reducing acrylamide levels in foods, and many studies have been conducted on different foods. In studies within this scope, many factors, such as the type and amount of ingredient in product formulations, the pH and moisture level of the product, the degree and duration of the heat applied for the production or consumption of the food, oil type and storage conditions were changed to reduce the level of acrylamide in foods [7,12,21,22,23,24,25,26,27]. In addition, the acrylamide level in foods were tried to be reduced before thermal process by using many traditional and modern methods, such as immersing foods in various solutions and applying asparaginase enzyme, applying high pressure, heating in microwave ovens, using ultrasound and pulsed electric fields [7,27,28,29,30,31,32,33].
4. Dietary acrylamide exposure: It is very important to assess the level of exposure to consumers to acrylamide-contaminated foods in terms of understanding the potential health risks of acrylamide and developing new strategies for the future [34]. The European Commission and Joint FAO/WHO Expert Committee to Food Additives (JECFA) reported that studies on exposure and risk assessments arising from acrylamide-contaminated foods are not sufficient and there is a need for systematic and comprehensive studies in this context [35,36]. In this context, many countries and researchers conduct risk analyses for dietary acrylamide exposure and evaluate acrylamide in terms of health. In studies of dietary acrylamide exposure, human exposure to acrylamide from different foods was determined to be 0.43 µg/kg bw/day in Poland [37], 0.22 µg/kg bw/day in Turkey [38], and 0.38 µg/kg bw/day in Portugal [39]. EFSA (2015) stated that infants and children are the group most exposed to acrylamide (average acrylamide intake 0.5–1.9 µg/kg bw/day), and infant formulas have a significant share in this exposure [11]. In the same report, it was stated that the mean acrylamide exposure in young, adult, elderly and older groups ranged between 0.4 and 0.9 µg/kg bw/day.
5. The effect of acrylamide on health: Acrylamide, which is taken into the body through contact, digestion and respiration, is easily absorbed and dispersed in the body because it has a low molecular weight. The excretion of acrylamide and its compounds from the body is quite rapid. It was reported that acrylamide taken into the organism can bind to DNA, RNA and proteins in some tissues and cells by undergoing chemical reactions, and their desire and potential to form compounds with these substances is high [40,41,42,43]. Acrylamide was identified as a “possibly carcinogenic to humans” compound by the International Agency for Research on Cancer (IARC) in 1994 and classified in group 2A [44,45]. Acrylamide is predicted to be a human carcinogenic [46]. The European Commission has classified acrylamide in Category 1B as a carcinogen and mutagen, and in Category 2 as a reproductive toxicant [35]. The European Chemical Agency (2022) included acrylamide in the List of Substances of Very High Concern [47]. The widespread use of acrylamide as a synthetic chemical and the surprising discovery that it occurs naturally in foods raises a complex problem. Is the presence of acrylamide in foods risky for consumers’ health?

In pre-natal exposure studies on humans, it was found that acrylamide taken during pregnancy was transferred to the placenta [48] and the increased acrylamide exposure caused lower fetal development [49,50]. It was also demonstrated in many studies that acrylamide exposure can significantly affect neurological changes in humans [45,51,52,53,54]. Although acrylamide was shown to be mutagenic, genotoxic, immunotoxic and carcinogenic in experimental animals [55,56], the results of epidemiological studies investigating the relationship between dietary acrylamide exposure and cancer in the general population in humans were inconsistent [45,57]. 

Cancer is the second most common disease after cardiovascular diseases among the causes of death caused by diseases today [58,59]. In a study on global cancer statistics, Sung et al. (2021) reported that there were 19.3 million cancer cases in 2020, 9.9 million of which resulted in death, and estimated that there would be 28.4 million cancer cases in 2040 [60]. 

Acrylamide, whose relationship with cancer was studied, is present in different levels in many foods frequently consumed in our daily diet. In addition, dietary acrylamide exposure continues throughout life, depending on changing consumption habits. The biggest concern about acrylamide is that the health risks have not been fully clarified yet. In the literature, it is possible to see studies that systematically evaluate epidemiological studies investigating dietary acrylamide exposure and cancer risk. However, unlike similar systematic reviews, in this study, more details were discussed such as the country where the study was conducted, the age ranges of the participants, the types of questionnaires used in the study, the types of questions included in the questionnaires, and the percentage of contribution of foods to acrylamide exposure. In addition, epidemiological studies examining the relationship between dietary acrylamide exposure and cancer were examined in detail in terms of both content and study types, and dietary acrylamide exposure was evaluated according to cancer type, related organs and systems, in this study. Again, in this study, meta-analysis studies, which are not discussed in other systematic reviews, but whose importance has increased in recent years, are included.

## 2. Materials and Methods

### 2.1. Search Strategy

The epidemiological studies published between January 2002 and April 2022 examining the relationship between dietary acrylamide exposure and cancer constitute the material of this study. Three authors (B.B., B.Ç. and G.K.) conducted search databases (PubMed, Isı Web of Knowledge, Scopus, Science Direct) using with the following keywords and combinations: Dietary OR (Diet) Acrylamide AND Cancer, Acrylamide Intake AND Cancer AND Risk, Acrylamide Exposure AND Cancer AND Risk. All relevant studies were systematically reviewed in accordance with the PRISMA guidelines [61].

### 2.2. Inclusion and Exclusion Criteria

After assessing the results, articles were considered eligible if they investigated the relationship between human cancer risks and acrylamide. The review was limited to studies on dietary exposure to acrylamide. 

Articles related to transplacental or environmental exposure, articles with synthetic AA, articles describing animal experiments, and tissue research and literature reviews were excluded from the study. Additionally, the articles written in languages other than English were excluded. 

**Figure 1:**
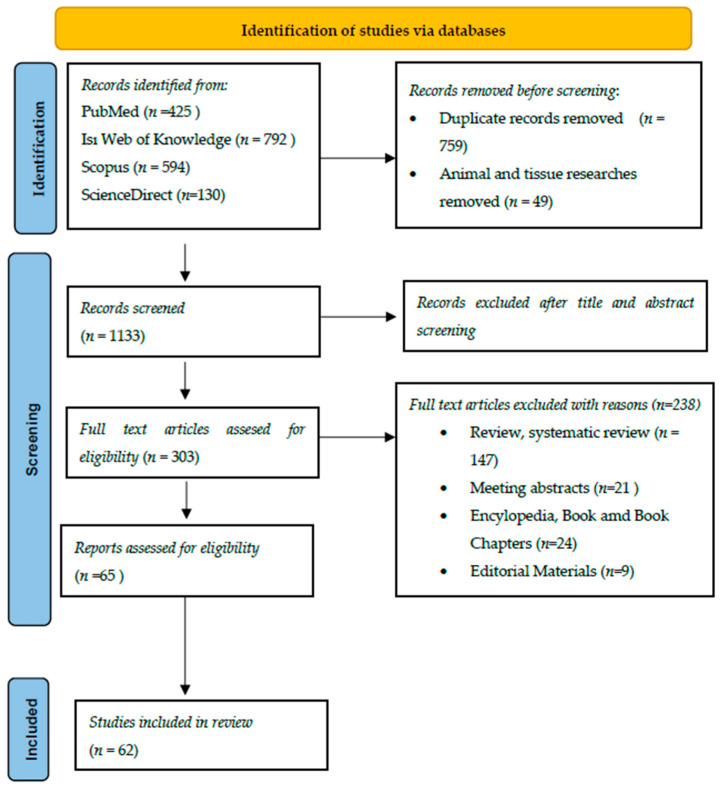
Data extraction.

The method differences of the studies were not accepted as an exclusion criterion of the study. However, systematic review and meta-analysis studies were included, while only systematic reviews were not included in the study (Figure 1).

### 2.3. Data Extraction

Data were extracted and verified using an excel table designed by the authors. The data collected included author names, publication years and full names, and duplicated articles were excluded in this way.

We abstracted the studies’ objectives, methods, sample group and sizes, countries, main outcomes, the type of cancer investigated (lung, prostate, uterus, pancreas etc.), the source from which acrylamide information is obtained, and the main food source where acrylamide is taken into the body for each study. In accordance with the purpose of the study, we grouped the obtained data according to the type of study and the site-specific cancer.

## 3. Results

### 3.1. First Part

Epidemiological studies examining the relationship between dietary acrylamide exposure and cancer risk were grouped chronologically under four headings: prospective cohort, case cohort, case control, and systematic review—meta-analysis studies.

#### 3.1.1. Prospective Cohort Studies 

The Women’s Lifestyle and Health Cohort study was conducted on 43,404 women (mean age 39–49% postmenopausal) in Sweden between 1991 and 2002. The aim of this study by Mucci et al. (2005) was to examine the relationship between dietary acrylamide exposure and breast cancer [62]. Nutritional information was obtained through a semi-quantitative Food Frequency Questionnaire (FFQ), which includes information on coffee, French fries, bread, biscuits, crackers, breakfast cereal, pancakes and meatballs, which are known to be rich in acrylamide. The acrylamide levels of foods were taken from the Swedish National Food Authority (SNFA) database (Table 1).

Another study (The Swedish Mammography Cohort) was conducted on 61,467 women (41–73 years old) in Sweden between 1987 and 2003 [90]. In this study, the relationship between dietary acrylamide exposure and colorectal cancer was examined. Acrylamide levels in this study were taken from the SNFA and the US Food and Drug Administration (FDA) database, and the dietary acrylamide exposure was calculated by multiplying the data obtained from the semi-quantitative FFQ (includes information on 67 foods commonly consumed in Sweden) (Table 2).

A study (Danish Diet, Cancer and Health Cohort) was conducted to investigate the effect of nutrition on cancer risk on 29,875 people aged 50–64 years in Denmark between 1993 and 1997. Olesen et al. (2008) examined the relationship between dietary acrylamide exposure and breast cancer, taking the data from this study as a reference [65]. Nutritional information and habits of individuals were determined by FFQ. The acrylamide level was determined by measuring 30 mL of blood taken from individuals in LC-MS with Hb-AA and Hb-GA biomarkers (Table 1).

Three studies (The Swedish Mammography Cohort) were conducted in Sweden that included data from 1987–1997 and examined the associations between cancer cases in the reproductive system and dietary acrylamide exposure. In these studies, 61,433 individuals for breast cancer [66], 61,226 individuals for uterine cancer [77], and 61,057 individuals for ovarian cancer [85] were examined. The same researchers also examined the relationship between dietary acrylamide exposure and colorectal cancer [98] and prostate cancer [105] in 45,306 men (45–79 years of age) between 1998 and 2007 (Cohort of Swedish Men). Nutritional information about the amount and frequency of food consumption of individuals was determined by FFQ to shed light on 1 year ago. Acrylamide levels of foods were taken from the SNFA database (Table 1, Table 2 and Table 3).

A study (Nurses’ Health Study II Cohort) was conducted on 90,628 healthcare professionals in the United States between 1991 and 2005. Wilson et al. (2009) examined the relationship between dietary acrylamide exposure and breast cancer using data from this study [67]. The age range of the professionals included in the study was 25–42. Nutritional status of individuals has been determined by the FFQ, which is carried out every 4 years in 1991, 1995, 1999 and 2003 and reflects the nutritional status of the previous year. The prepared FFQ contains information on more than 130 foods. Frequency of consumption of foods was taken according to nine possible answers ranging from “never” to “6 or more per day”. For the calculation of dietary acrylamide exposure, 42 foods known to be rich in acrylamide levels were included in the study. Acrylamide levels of foods were taken from the FDA and SNFA databases (Table 1).

Between 1980 and 2006, a study was conducted on pre- and postmenopausal health workers (Nurses’ Health Study Cohort I) in the United States. Wilson et al. (2010) examined the association between dietary acrylamide exposure and breast cancer (88,672 individuals), uterine cancer (69,019 individuals), and ovarian cancer (80,011 subjects), using data from the Nurses’ Health Study Cohort I [69]. In the study, smokers and non-smokers were examined separately. The age range of individuals was 30–55. Sociodemographic characteristics and nutritional information of individuals were determined by the FFQ applied every 4 years (61–116 foods included). Frequency of consumption of foods was taken according to nine possible answers ranging from “never” to “6 or more per day”. Acrylamide levels of foods were taken from the FDA and SNFA databases (Table 1). 

Hirvonen et al. (2010) examined the association between dietary acrylamide exposure and cancer risks in Finnish smoking men [96]. The study was conducted on 27,111 people aged 50–69 years who voluntarily participated in the Alpha-Tocopherol, Beta-Carotene Cancer Prevention (ATBC) study without a history of cancer and who smoked at least five cigarettes per day. As a result of the research, a total of 2958 cancer cases with different types of cancer were identified. Initially, a pilot study was conducted in which 190 men participated and the nutritional method was tested. Consumption amount and frequency of foods were determined by FFQ (the frequency of consumption of foods was determined as month, week and day) which reflects the nutritional information of the individuals for the previous year and includes 276 foods. The FFQ was given to the participants, and responses were received 2 weeks later. Acrylamide levels of foods were taken from Finnish data. The main sources of dietary acrylamide exposure were coffee (15.2 μg/day), French fries (5.47 μg/day), and rye bread (5.26 μg/day).

In another study conducted by Wilson et al. (2012), data from “The Health Professionals Follow-up Study (HPFS)” were used [108]. HPFS was performed on 47,896 healthcare workers aged 40–75 years in the United States between 1986 and 2006. The aim of the study was to examine the relationship between dietary acrylamide exposure and prostate cancer. Individuals’ dietary exposure to acrylamide was determined by the FFQ (containing information on more than 130 foods) conducted every 4 years. The frequency of consumption of foods was taken according to nine possible answers ranging from “never” to “6 or more per day”. Acrylamide levels of foods were obtained from FDA (Table 3).

Nutrition and life styles of individuals (The European Prospective Investigation into Cancer and Nutrition Cohort-EPIC) were examined in 10 countries in Europe (France, Italy, Spain, England, Netherlands, Greece, Germany, Sweden, Denmark, Norway) between 1992 and 1998. A total of 521,330 people participated in the EPIC surveys. In all, 367,903 of the participants were women. Then, 477,308 individuals were followed up in terms of pancreatic cancer between 1992 and 2003 (Table 3) [100], 301,113 individuals were followed up in terms of uterine cancer (Table 1) [87] and 325,006 individuals were followed up in terms of ovarian cancer between 2004 and 2010 (Table 1) [78], and the relationship between dietary acrylamide exposure and cancer types was examined. Nutritional acrylamide exposure was obtained from FFQ data (24-h dietary recall were bread, crispbread, rusks, coffee, potatoes, cakes, biscuits, and cookies) and information showing acrylamide levels of foods registered in the European Union database (European Commission Institute for Reference Materials and Measurements). 

Lujan-Barroso et al. (2014) examined the association between dietary acrylamide exposure and esophageal adenocarcinoma, and esophageal squamous cell carcinoma, tumors of esophageal cancer and esophageal cancer, based on the EPIC data (Table 2) [93]. 

Using data from a prospective cohort (Mr. and Ms. OS Hong Kong study) of 4000 men and women over 65 years of age in China from 2003 to 2014, the relationship between dietary acrylamide exposure and cancer types was investigated [104]. The nutritional information of individuals was determined by FFQ (contains information on 329 foods). The acrylamide level of foods was calculated using data from the 1st Hong Kong Total Diet Study database. In the study, foods that contributed the most to dietary acrylamide exposure were determined as fried vegetables and products (43.7%), and cereals and cereal products (28.9%) (Table 2, Table 4 and Table 5).

Lipunova et al. (2017) conducted a prospective cohort study by following 5000 men and women randomly sampled from the Netherlands Cohort Study, which started in 1986, and in which 120,852 people participated, for 17.3 years [122]. The aim of the study was to examine the relationship between dietary acrylamide exposure and cutaneous malignant (CMM), superficial spreading (SSM) and nodular melanoma (NM) (Table 5). Information on the habits of individuals, the amount and frequency of food consumption were determined by FFQ (consisting of 150 foods). Acrylamide levels of foods were obtained from the Dutch Food and Consumer Product Safety Authority database (Table 5).

Graff et al. (2018) examined the relationship between dietary acrylamide exposure and renal cell carcinoma using data from the prospective Health Professionals Follow-up Study (HPFS, 51,529 male health professionals, ages 40–75) and Nurses’ Health Study (NHS, 121,701 female nurses, ages 30–55) studies performed in the United States between 1986–2014 and 1980–2014, respectively [113]. The study included 47,797 men and 88,767 women who were not initially diagnosed with cancer, and the individuals were followed for more than 20 years. Dietary acrylamide exposure was calculated based on 46 acrylamide-containing foods reported in food frequency questionnaires completed every 4 years (Table 3).

McCullough et al. (2019) followed the diet, lifestyle, and cancer risk factors of 102,154 men and women through 30 June 2013, based on the Cancer Prevention Study-II Nutrition Cohort data, updated with new exposure information in 1999, conducted in the United States [114]. The relationship between dietary acrylamide exposure and renal cancer was investigated in the study. Nutritional information of individuals was determined by FFQ (152 items). In the FFQ, respondents were asked how often, on average, they had consumed each listed item in the past year. Frequency categories ranged from “never/rarely” to “4+ times a day” for food and “never/rarely” to “6+ times a day” for beverages. The foods that contributed the highest to dietary acrylamide exposure were French fries (23%), coffee (15%), and bread (10%), respectively (Table 3).

A population-based prospective cohort study (The Japan Public Health Center-JPHC Study) was conducted in Japan between 1990 and 2013. The JPHC Study was conducted in two phases and covered a total of 11 regions. While Cohort I included Iwate, Akita, Nagano, Okinawa-Chubu, and Tokyo, Cohort II included the Ibaraki, Niigata, Kochi, Nagasaki, Okinawa-Miyako, and Osaka regions. 140,420 (68,122 men-71,698 women) individuals between the ages of 40–69 participated in this research. Nutritional status of individuals was determined every 5 years through the FFQ, which includes information on 138 foods and beverages. Participants were asked for nine possible responses for both eating frequency (never, 1–3 times/mo, 1–2 times/wk, 3–4 times/wk, 5–6 times/wk, 1 time/d, 2–3 times/d, 4–6 times/d, or ≥7 times/d) and drinking frequency (<1 cup/week; 1–2 cups/week; 3–4 cups/week; 5–6 cups/week; 1 cup/day; 1–3 cups/day; 4–6 cups/day; 7–9 cups/day; or ≥10 cups/day). In addition, there were three possible answers to determine the amount of consumption (less than half the standard portion size, standard portion size, or more than 1.5-fold the standard portion size) [123]. A total of eight studies were conducted using the JPHC Study database.

Kotemori et al. (2018) examined the association of dietary acrylamide exposure with breast (48,910 women), and uterine and ovarian cancers (47,185 women) [73,83]. Acrylamide levels of foods were obtained from reports showing acrylamide levels of commonly consumed foods in Japan. In the study, the foods that contributed the most to acrylamide exposure were determined as coffee 24%, green tea 23%, biscuits and cookies 13%, potato products 13%, and vegetables 11% (Table 1).

Liu et al. (2019) examined the relationship of dietary acrylamide exposure with esophageal, gastric and colorectal cancer. In the study, which included 87,628 participants (40,732 men and 46,896 women), individuals were followed for an average of 15.5, 15.3 and 15.3 years for esophageal, gastric and colorectal cancer, respectively [94]. Acrylamide levels of foods were obtained from reports showing acrylamide levels of commonly consumed foods in Japan. In the study, the food and beverages that contributed the most to acrylamide exposure were determined as coffee 28%, green tea 22%, biscuits and cookies 11%, potato products 11% and vegetables 11% (Table 2). Another study by Liu et al. (2020) on this subject was carried out in 2020, with a total of 85,503 people, including 39,982 men and 45,321 women [118]. The relationship between dietary acrylamide exposure and lung cancer risk was examined in the study. In the study, in men, 379 cases were recorded as adenocarcinoma, 323 cases were recorded as squamous cell carcinoma, 140 cases were recorded as small cell lung carcinomas, and 345 cases were recorded as unclassified cases. In women, 358 adenocarcinomas and 127 unclassified cases were recorded. The main contributors to dietary acrylamide exposure were identified as coffee (men: 32.0%, women; 23.6%), green tea (men: 20.2%, women; 22.8%), potatoes and cereals (men: 10.4%, women; 12.8%), biscuits and cookies (men: 8.0%, women; 12.8%), and vegetables (men: 9.9%, women; 11.5%) (Table 4). 

Kito et al. (2020) examined the relationship between dietary acrylamide exposure and pancreatic cancer risk [102]. A total of 89,729 people, including 42,071 men and 47,658 women, participated in the study. In the study, which lasted an average of 15.2 years, acrylamide levels of foods were obtained from reports showing acrylamide levels of commonly consumed foods in Japan. The food groups that contributed the most to dietary acrylamide exposure were beverages (total 53%; 28% for coffee, 21% for green tea, 2% for beer, and 2% for others), followed by confections (total 16%; 11% for biscuits/cookies, 3% for chocolate, and 2% for others), vegetables (total 11%; 3% for sweet pepper, 3% for onion, 3% for bean sprouts, and 2% for others), and potatoes (11%) (Table 2).

In recent studies using the JPHC Study database, people recorded in 1995–1998 were followed for various types of cancers for approximately 16 years (85,305 people with liver cancer [103], 85,303 people with hematological malignancies (39,982 men and 45.321 women) [121], 88,818 people with renal cell, prostate, and bladder cancers (41,534 men and 47,284 women) [110]. A total of 2823 cancer cases were identified in these studies. Nutritional exposure to acrylamide was calculated from FFQ data (average consumption frequency of 138–147 foods and beverages in the last year) and reports showing acrylamide levels of commonly consumed foods in Japan. The foods that contributed the most to dietary acrylamide exposure were determined as coffee, green tea, potatoes and starches, vegetables, and biscuits and cookies, respectively (Table 3, Table 4 and Table 5).

#### 3.1.2. Case Cohort Studies

The case cohort studies examining the associations between dietary acrylamide exposure and cancer risk were the Netherlands Cohort Study and the United Kingdom Women’s Cohort. A total of 13 publications were made from these two case cohort studies. Between 1986 and 1997, research on nutrition and health (The Netherlands Cohort Study) was conducted on 120,852 people (58,279 men, 62,573 women: 55–69 years old) in the Netherlands. Hogervorst et al. (2007) examined the relationship of dietary acrylamide exposure with ovarian, uterine, and breast cancer by reference to the Netherlands Cohort Study data (Table 1) [64]. The same researcher also examined the relationship of dietary acrylamide exposure with gastrointestinal system cancers (Table 2) [91], urinary system cancers (Table 3) [106], lung cancer (Table 4) [117] and brain cancer (Table 5) [119] in individuals followed between 1986 and 2002. They also investigated the relationship of dietary acrylamide exposure with uterine cancer (Table 1) [80] and ovarian cancer (Table 1) [88] in individuals followed between 1986 and 2006. Hogervorst et al. (2019) also examined the relationship between dietary acrylamide exposure and colorectal cancer in terms of Kirsten-ras (KRAS) and the relationship between dietary acrylamide exposure and ER+ breast cancer in terms of genetic variants (Table 1) [74]. In the study, 57 genes and two gene deletions in single nucleotide polymorphisms (SNPs) were examined as genetic variants. The consumption amount, frequency and habits of foods were determined by the FFQ, which includes information on 150 foods. Acrylamide levels of foods were obtained from a study conducted in 2002 by the Dutch Food and Consumer Product Safety Authority. In the study, the foods that contributed the most to acrylamide exposure were determined as potato chips 31%, French fries 21%, spicy special cakes 16%, coffee 13% and bread 10%.

Schouten et al. (2009) investigated the relationship between dietary acrylamide exposure and cases of oral cavity, oro-hypopharynx, larynx (Table 4), and thyroid cancer (Table 5) [116]. Bongers et al. (2012) investigated the relationship between dietary acrylamide exposure and Lymphatic Malignancies (chronic lymphocytic leukemia, diffuse large cell lymphoma, Waldenstrom macroglobulinemia and immunocytoma, follicular lymphoma, mantle cell lymphoma, T-cell lymphomas) (Table 5) [120]. Pedersen et al. (2010) examined the association between dietary acrylamide exposure and estrogen receptor (ER) + breast cancer and progesterone receptor (PR) + breast cancer [68]. Perloy et al. (2018) examined the relationship between dietary acrylamide exposure and prostate cancer [109]. All four researchers based data of acrylamide intake and level on the Netherlands Cohort Study.

The dietary habits of 35,372 women (35–69 years old) in England between 1995 and 1998 were determined within the scope of the United Kingdom Women’s Cohort study by means of the FFQ, which contains information about 217 foods. Considering the consumption data of this study, Burley et al. (2010) examined the relationship between dietary acrylamide exposure and pre- and post-menopausal breast cancer (Table 1) [70]. In all, 24 foods were included in the dietary acrylamide exposure calculation. Acrylamide levels of foods were obtained from previous studies by the European Union (European Commission Institute for Reference Materials and Measurements) and Food Standards Agency. The foods that contributed the most to total daily acrylamide exposure were potato chips (28%), bakery products (17%), potato crisps (14%), bread (10%) and coffee (8%), respectively.

#### 3.1.3. Case Control Studies

Case control studies were the first to examine the relationship between dietary acrylamide exposure and cancer types. In this context, 11 publications were reached. 

The first study to examine the relationship between dietary acrylamide exposure and cancer was conducted by Mucci et al. (2003) in Sweden [97]. The study was a population-based study and was conducted with individuals who were born in Sweden between 1918–1942 and resided in Stockholm for at least 1 month between November 1992 and December 1994. The aim of the study was to examine the relationship between dietary acrylamide exposure and bladder, renal (Table 3) and colorectal cancer (Table 2). The number of individuals in the control group was determined as 538. In the study, the retrospective 5-year dietary habits of individuals were determined by a semi-quantitative FFQ. The FFQ included 180 food-related information and 10 possible consumption frequency responses (ranging from never to 2–3 times a day). Acrylamide analysis was performed on more than 100 food products. According to the results of acrylamide analysis, foods were divided into four groups as 0–100 µg/kg, 100–200 µg/kg, 200–600 µg/kg and 600 µg/kg and above/more.

Mucci et al. (2004) examined the relationship between dietary acrylamide exposure and renal cancer [111]. The research was a population-based case/control (379/353) study on individuals aged 20–79 years residing in Sweden between June 1989 and December 1991. Information on the nutrition of individuals before 1987 was obtained by face-to-face interviews through the FFQ. Consumption habits related to 11 foods, including products such as coffee, French fries, biscuits and bread, were determined. Acrylamide levels of foods were taken from SNFA and FDA databases.

Pelucchi et al. (2006) conducted a case/control study based on hospital data in Southern European countries, Italy and Switzerland, between 1991 and 2002 [63]. The aim of the study was to examine the relationship of dietary acrylamide exposure with breast and ovary cancer (Table 1), esophagus and large bowel cancer (Table 2), prostate cancer (Table 3), and oral cavity, pharynx and larynx cancer (Table 4). Breast cancer, esophageal cancer, oral cavity/pharynx cancer, ovarian cancer, large bowel cancer, larynx cancer and prostate cancer findings were obtained from data belonging to 1991–2001, 1992–1999, 1991–1997, 1992–1999, 1992–2001, 1992–2000 and 1991–2002, respectively. The number of cases/control groups (mean age) in each cancer type was determined to be 2900/3122 (55 age), 1395/1066 (60 age), 749/1772 (57 age), 1931/2411 (56 age), 2280/4765 (63 age), 527/1297 (61 age) and 1294/451 (66 age), respectively. The same investigator also examined the relationship between dietary acrylamide exposure and renal cancer (Table 3) in 2007. This research was carried out in Italy between 1992 and 2004 (cases 767/controls 1534) [112]. Nutritional information was determined through the FFQ, which includes information on 78 foods. The acrylamide level of foods was taken from the World Health Organization and the French Agency for Food Safety database. The foods that contributed the most to acrylamide exposure were fried/baked potatoes (29.6%), white bread (28.6%), biscuits (15%), coffee (12.4%) and crackers (6.5%).

Wilson et al. (2009) conducted a population-based study in Sweden between 2001 and 2002 (The Cancer of the Prostate in Sweden-CAPS) and examined the relationship between dietary acrylamide exposure and prostate cancer in 1499 cases/1118 control group members aged 35–79 years (Table 3) [107]. The nutritional information of individuals was obtained through a FFQ (contains information on 261 foods) that shed light on 1 year ago. However, 18 foods were included in the study to determine acrylamide exposure. Acrylamide levels of foods were taken from the SNFA database (fried potatoes (292 µg/kg), chips and popcorn (744 µg/kg), ground meat dishes (64 µg/kg), cereal (184 µg/kg), crispbread (rye) (527 µg/kg), crispbread (wheat) (93 µg/kg), black pudding (40 µg/kg), buns and cookies (115 µg/kg), pancakes (21 µg/kg), sausage (40 µg/kg), fish sticks (30 µg/kg), coffee 25 µg/kg), bread made of rye (122 µg/kg), crackers (205 µg/kg) and muesli (31 µg/kg)). An analysis of acrylamide adducts to hemoglobin, an indicator of acrylamide exposure, was conducted in blood samples from 170 cases and 161 control participants. Acrylamide exposure calculated through a FFQ and acrylamide adduct levels were compared using partial Pearson correlation coefficients.

Pelucchi et al. (2011) examined the relationship between acrylamide exposure and pancreatic cancer in Italy between 1991 and 2008 (Table 2) [99]. Between the mentioned years, 326 cases (174 men-152 women, 34–80 years old, mean age: 63) were detected. The control group consisted of 652 individuals. Nutritional information and habits were determined by the FFQ, which determined 2 years before the diagnosis of pancreatic cancer. The FFQ contained information on 78 foods. Foods were divided into six groups: (i) bread, cereals and first courses; (ii) second courses (i.e., meat, fish and other main dishes); (iii) side dishes (i.e., vegetables); (iv) fruits; (v) sweets, desserts and soft drinks; and (vi) milk, hot beverages and sweeteners. The acrylamide level of the foods was taken from the World Health Organization and Agence Francaise de Se’curite´ Sanitaire des Aliments database. Contributions of food to the total acrylamide exposure were 27.2% for fried/baked potatoes, 25.5% for white bread, 17.1% for sweet biscuits and 11.3% for coffee.

Another population-based study conducted in Sweden between 1994–1997 was carried out by Lin et al. (2011) [92]. The aim of the study was to examine the relationship of dietary acrylamide exposure with esophageal cancer and esophageal cancer tumors such as esophageal adenocarcinoma, gastroesophageal junctional adenocarcinoma, and esophageal squamous cell carcinoma (Table 2). The control group (820 people) was randomly selected according to age, gender and frequencies. Nutrition information was determined by the FFQ, which consists of 63 foods and beverages, shedding light on 20 years retrospectively. French fries, fried potatoes, baked potatoes, soft bread, soft coarse bread, crisp bread, biscuits, cookies and coffee were included in the exposure assessment. The acrylamide level of the foods was taken from the SNFA database. The main foods contributing to dietary acrylamide intake were coffee (31%), French fries (22%), soft coarse bread (12%), soft bread (11%), crispy bread (8%), French fries (7%), baked potatoes (6%), and biscuits/cookies (3%).

Xie et al. (2013) examined the relationship of ovarian cancer with hemoglobin adducts of acrylamide (Hb-AA) and the glycidamide adducts of acrylamide (Hb-GA) [86]. Information on individuals’ exposure to acrylamide was obtained from the NHS I (1990) and NHS II (1999) prospective cohort studies. Hb-AA and Hb-GA measurements were performed by HPLC/MS/MS (Table 1).

A study based on the EPIC data was conducted on 385,747 people living in eight countries in Europe (France, Italy, Spain, England, Netherlands, Greece, Germany, Sweden) between 2005 and 2010. The aim of the study was to examine the relationship of uterine cancer with hemoglobin adducts of acrylamide (Hb-AA) and glycidamide adducts of acrylamide (Hb-GA) in non-smoking postmenopausal women (Table 1). In all, 383 cases of uterine cancer, 171 of which were type I, were detected. The number of individuals in the control group was 383. Hb-AA and Hb-GA measurements were performed by HPLC/MS/MS. In the study, the relationship of four types of exposure, namely Hb-AA, Hb-GA, their sum (HbAA/HbGA) and their ratio (HbGA/HbAA,) with uterine cancer was investigated [81].

Pelucchi et al. (2016) examined the relationship between dietary acrylamide exposure and endometrial cancer risk in a case-control study conducted in three regions of Italy (the provinces of Pordenone and Milan, in Northern Italy, and the provinces of Naples, in Southern Italy) between 1992 and 2006 [82]. The study was carried out on 454 cases (18–79 years) and 908 controls (19–79). A FFQ during the stay of cases and controls was used to assess usual nutrition during the two years prior to diagnosis (or admission to hospital for controls). The FFQ was divided into six sections and was prepared to include 78 food groups and their recipes. At the end of each section, an open-ended question was used to describe other foods eaten at least once a week. In addition, an additional section on the consumption of alcoholic beverages was created in the survey. Participants were asked to indicate the average weekly frequency of consumption for a food and their normal portion sizes. The main foods contributing to the total acrylamide exposure were fried/baked potatoes (28%); white bread (24%); cookies (16%); coffee (14%); crackers, breadsticks, and melba toast (10%).

Pelucchi et al. (2017) investigated the relationship between pancreatic cancer and dietary acrylamide exposure under the International Pancreatic Cancer Case-Control Consortium (PANC4) based on six case-control studies (USA, Italy, Australia) (Table 2) [101]. In this context, 1975 cases (4239 controls) were identified. Nutritional information was obtained from FFQ and acrylamide levels of foods from international databases. Foods included in the calculation of acrylamide exposure: coffee, breads, potato products, various breakfast cereals, biscuits and cookies, gingerbread and spiced cakes, chocolate products (such as brownies and candy bars), several types of snacks and pastries, fried fish, fried chicken, pizza, tacos, fried rice and beer.

#### 3.1.4. Meta-Analysis Studies

This section included publications that systematically compile the relationship between dietary acrylamide exposure and cancer risk, as well as analyzing the data obtained through meta-analysis technique and producing new results. In this context, 10 publications were reached. 

Pelucchi et al. (2011) analyzed 25 studies published in Medline and PubMed databases until 2009 using meta-analysis technique. In the study, the relationship between dietary acrylamide exposure and 10 different cancer types was evaluated [71].

Je (2015) analyzed data from four prospective cohort studies (the Netherlands Cohort Study, NLCS; the Nurses’ Health Study, NHS; the Swedish Mammography Cohort, SMC; and the European Prospective Investigation into Cancer and Nutrition, EPIC cohort) published up to 2014 and evaluated the relationship between uterine cancer and dietary acrylamide exposure [79]. A total of 453,355 participants were included in the study (Table 1).

Pelucchi et al. (2015) reviewed 32 studies examining the relationship between dietary acrylamide exposure and the risk of endometrial cancer published between 2009 and 2014 [72]. Huang and Wang (2019) systematically reviewed some of the studies published in PubMed, Medline and Embase databases until February 2017 on this subject and evaluated them using the meta-analysis technique (Table 1, Table 2, Table 3, Table 4 and Table 5) [84].

Adani et al. (2020) systematically reviewed studies published up to 25 February 2020 to examine the relationship of dietary acrylamide exposure with breast, endometrial, and ovarian cancer risk [75]. They evaluated a total of 18 articles, 16 of which were cohort and two of which were case-control studies, using the meta-analysis technique (Table 1).

Atabati et al. (2020) searched databases such as Web of Science, Scopus, PubMed, Embase until September 2019 [42]. In their study, in which they reached a total of 57 publications, they evaluated the relationship between dietary acrylamide exposure and breast cancer (Table 1).

Jiang et al. (2020) evaluated eight epidemiological studies of dietary acrylamide exposure and renal cell carcinoma risk in the PubMed, EMBASE, and Cochrane databases through the meta-analysis technique [115]. On the other hand, Benisi-Kohansal et al. (2021) systematically examined the PubMed, ISI Web of Science and Scopus databases until August 2020 and evaluated the relationship of dietary acrylamide exposure with breast, endometrial and ovarian cancer risk using meta-analysis technique in a study that included 14 cohort studies (Table 1) [76].

In a study examining the relationship between nutrition and ovarian cancer risk, Khodavandi et al. (2021) systematically examined the databases of Scopus, PubMed and Wiley Online Libraries until 24 November 2019 and evaluated the data obtained through meta-analysis technique (Table 1) [89].

Filippini et al. (2022) systematically reviewed the epidemiological studies searched in PubMed, Scopus, and Web of Science databases until 7 March 2022 and reached a total of 16 publications [95]. In the study, the researcher examined the relationship between dietary acrylamide exposure and site-specific cancer types using the meta-analysis technique (Table 2, Table 3, Table 4 and Table 5).

### 3.2. Second Part

In this section, studies examining the relationship between dietary acrylamide exposure and cancer risk were classified systematically as reproductive system (Table 1), gastrointestinal system (Table 2), urinary system (Table 3), respiratory system (Table 4) and other systems (Table 5), and the results were presented in tables according to the systems in which the organs are located.

## 4. Discussion

### 4.1. First Part

In studies examining the relationship between dietary acrylamide exposure and cancer risk, the most preferred method was prospective cohort. Case-control and case-cohort studies were limited. On the other hand, there has been an increase in studies in which systematic review-meta-analysis techniques are used in recent years. Studies examining the relationship between dietary acrylamide exposure and cancer risk have been mostly conducted in Sweden and Japan. Over 1 million people participated in only prospective cohort studies. The European Prospective Investigation into Cancer and Nutrition Cohort (EPIC) was the largest prospective cohort study in terms of both the area it covers and the number of participants. In case cohort studies, the number of studies based in the Netherlands was high. When all studies were evaluated together, it was understood that researches were generally conducted in developed countries. In underdeveloped and developing countries, studies investigating dietary acrylamide exposure and cancer risk were scarce; therefore, new studies covering other countries are needed.

Although there were some case control studies in which dietary acrylamide exposure in individuals was determined by measuring biomarkers such as Hb-AA and Hb-GA, acrylamide exposure in studies was calculated using FFQ and databases showing acrylamide levels of various foods [65,81,86]. It should be noted that the acrylamide level of the foods included in the databases and represented in the FFQ may differ due to/in terms of features such as raw material properties and production method. While some FFQs contained fewer foods, others had more. In addition, the brands of foods, the frequency of consumption and the amount of consumption were also different from each other. Cooking methods that could significantly affect acrylamide levels were omitted for most foods. Therefore, although there were some similarities between the studies, a standard procedure could not be established in the methods used in the studies [90,96,104,108,114,122]. In addition, FFQs containing many questions may reduce the motivation of the interviewee or may be a potential source of bias. Therefore, determining the acrylamide level in foods using a typical FFQ brings some uncertainties [124]. In this context, Lin et al. (2011) calculated acrylamide exposure by ranking some foods that are thought to have high acrylamide formation, but they had difficulty comparing their results with other studies [92]. It is known that the food industry is in constant development and introducing new products to the market. Again, many factors, including the COVID-19 pandemic, affect consumers’ eating habits. When all these factors are considered, the calculation of dietary acrylamide exposure becomes more difficult.

Some studies highlighted that acrylamide exposure could have been caused an increased risk of cancer in non-smokers [64,65,75,79,84,92,116,122]. However, smoking plays a role in the conversion of acrylamide to glycidamide, a genotoxic and carcinogenic metabolite, through saturated enzymes [125]. Therefore, the increase in cancer risk with dietary acrylamide exposure in non-smokers requires careful interpretation. In addition, in a study conducted with only smokers in Finland [113], it was concluded that the risk of lung cancer increased with dietary acrylamide exposure. These conflicting results suggest that smoking is a confounding factor, particularly in studies derived from dietary acrylamide exposure. Because of the potential for smoking to increase dietary acrylamide exposure, findings from individuals who smoke need to be further investigated.

### 4.2. Second Part

In the current study, it was determined that the results of 63 epidemiological studies investigating the relationship between dietary acrylamide exposure and cancer risk differ from each other. For this reason, it is not correct for now to make an inference that a certain level or higher acrylamide exposure increases the risk of cancer in any organ or system-specific cancer type. However, studies showed that dietary exposure to acrylamide was lower in Eastern Asian countries, such as Japan and China, compared to European countries [73,83,94,102,103,104,110], reveal that the highest exposure was in the European region countries [78,81,92,112]. It is thought that this situation arises from the differences in the beliefs, cultures and eating habits of the societies.

Studies examining the relationship between dietary acrylamide exposure and cancer have often focused on the reproductive system. The number of publications reached within the scope of this study was 63 and approximately 70% (43 publications) of this consisted of research on the reproductive system and organs. In the reproductive system organs, breast cancer cases have mostly been examined. Thirteen out of 16, 12 out of 14 and nine out of 13 studies did not find a direct positive association between dietary acrylamide exposure with breast cancer, uterine cancer and ovarian cancer, respectively. Although a direct relationship of dietary acrylamide exposure with breast, uterine and ovarian cancer has not been demonstrated, it has been noted that some factors (smoking, estrogen and progesterone hormone levels, premenopausal period, some genes) may increase the risk of cancer in the reproductive system, especially of ovarian cancer [64,65,68,70,74,78,80,88].

Studies on the gastrointestinal system organs focused on colorectal cancer (11 studies). While some studies examined the relationship between dietary acrylamide exposure and colorectal cancer as a whole, some divided it into colon and rectum. It is very difficult to talk about the increase in cancer risk with dietary acrylamide exposure in other cancer types except esophageal cancer (eight studies). As a matter of fact, no statistical relationship could be found regarding gastric (five studies) and pancreatic cancer (eight studies). However, the risk of esophageal cancer was found to be relatively higher in non-smokers and obese individuals [92,93]. Therefore, new studies should be planned for special groups such as obese individuals. In addition, the fact that only two studies have been conducted showing the relationship between dietary acrylamide exposure and liver cancer (two studies) shows that there is a need for new studies in this area [95,103]. 

In studies on urinary system organs, it is almost impossible to generalize that dietary acrylamide exposure increases the risk of cancer. Of the 17 studies conducted in this context, only Hogervorst et al. (2008) stated that there may be a risk in terms of renal cancer [106].

Studies in the respiratory systems have similarly failed to demonstrate a strong association between dietary acrylamide exposure and cancer risk. Three of four studies, all of four studies and four of five studies found no direct positive association between oral cavity/pharyngeal cancer, laryngeal cancer and lung cancer with dietary acrylamide exposure, respectively. However, the existence of some statistically significant studies with different results should be considered [116,117].

When all the studies are evaluated, it was understood that the number of studies on the effect of dietary acrylamide exposure on brain cancer (two studies), thyroid cancer (two studies) and skin cancer (one study) was quite limited [116,119,122].

## 5. Conclusions

Acrylamide, whose presence in foods was shared with the public in 2002 and whose importance has been increasing in our lives since then, still contains many unknowns in terms of its formation mechanism, its level in foods and its effects on health. This uncertainty is a major concern when considering the potential risks of acrylamide. Therefore, more information should be obtained about all the factors that may be caused by dietary acrylamide exposure, and by managing this information, meaningful policies for public health should be produced. The high level of acrylamide in foods such as French fries, bread, biscuits and coffee, which have an important place in our daily life and which we can easily reach and consume throughout life, is an important issue that should be considered for our health. Therefore, many researchers have discussed various aspects of acrylamide, and research is still ongoing today.

In the light of the information obtained in this study, it is quite difficult to say clearly that there is a positive relationship between dietary acrylamide exposure and cancer types. In addition, no direct relationship was found with dietary acrylamide exposure on organs or systems. The lack of a standardized measurement method used by researchers also prevents the studies from being comparable. In this context, it seems that more research is needed to say whether exposure to acrylamide based on nutrition is a risk factor for developing various types of cancer. In order to obtain reliable results in explaining the relationship between dietary acrylamide exposure and cancer, it is very important that future research includes more people and foods. In addition, factors such as physical inactivity, harmful alcohol use, dyslipidemia, obesity and different chronic diseases should be considered as parameters in studies. Additionally, databases should be established in order to evaluate dietary acrylamide exposure, and the accuracy of the information in the databases should be tested and revised according to the new information obtained. The relationship between dietary acrylamide exposure and cancer risk is unclear. It is necessary to develop new evaluation methods that detect acrylamide and other similar compounds that can lead to clinical pictures, which is the end point of the disease, such as cancer. In this respect, acrylamide and other similar compounds should be included in health policies, and researchers should be supported in terms of information and resources. In addition, legislation on foods with high levels of acrylamide should be developed.

## Figures and Tables

**Table 1:** Epidemiological studies examining the effect of dietary acrylamide exposure on reproductive cancer types.

Type of Cancer—Reference	Cases	Mean Dietary Acrylamide Intake	Risk
**Breast Cancer**			
Mucci et al. (2005) [62]	667	25.9 µg/day (0.37 µg/kg bw/day)	na
Pelucchi et al. (2006) [63]	2900	24.32 µg/day (0.38 µg/kg bw/day)	na
Hogervorst et al. (2007) [64]	1350	21.0 ± 11.9 µg/day (0.32 ± 0.19 µg/kg bw/day	na
Olesen et al. (2008) [65]	374	Hb-AA: 47 (pmol/g globin) Hb-GA: 26 (pmol/g globin)	No relationship was found between Hb-AA (p: 0.83) and Hb-GA (p: 0.65) levels and breast cancer. However, a positive correlation was found between ER+ positive breast cancer and Hb-AA in non-smokers.
Larsson et al. (2009) [66]	2952	24.6 ± 7.6 µg/day (0.38 ± 0.17 µg/kg bw/day	na
Wilson et al. (2009) [67]	1179	20.2 µg/day (0.32 µg/kg bw/day	na
Pedersen et al. (2010) [68]	1690	18.3 µg/day (0.27 µg/kg bw/day)	No relationship was found between breast cancer and dietary acrylamide exposure. However, in ER+, PR+ and ER+/PR+ breast cancer cases, a partial increase was detected with acrylamide exposure, which was not statistically significant.
Wilson et al. (2010) [69]	6301	9–26 µg/day (0.13–0.42 µg/kg bw/day)	na
Burley et al. (2010) [70]	1084	15 µg/day (0.23 µg/kg bw/day)	No relationship was found between breast cancer and dietary acrylamide exposure. However, a positive relationship was found between premenopausal breast cancer and acrylamide exposure.
Pelucchi et al. (2011) [71]	9048	-	na
Pelucchi et al. (2015) [72]	16,773	-	na
Kotemori et al. (2018) [73]	792	7.0 ± 3.7 µg/day (0.14 ± 0.13 µg/kg bw/day)	na
Hogervorst et al. (2019) [74]	844	20.6 ± 11.3 µg/day	No positive correlation was found between ER+ breast cancer and dietary acrylamide exposure. On the contrary, an inverse relationship was found between acrylamide and ER+ breast cancer. However, a significant association was found between several SNPs genes (rs1056827 in CYP1B1, rs2959008 and rs7173655 in CYP11A1, the GSTT1gene deletion and rs1052133 in hOGG1) and acrylamide exposure. It was stated that acrylamide may cause breast cancer with its effect on sex hormones.
Adani et al. (2020) [75]	-	-	No association was found between exposure and risk for breast cancer, particularly in never-smokers and postmenopausal women, or an inverse relationship was found. In a limited subgroup analysis of premenopausal women, acrylamide intake starting at 20 μg/day was associated with a linear increase in breast cancer risk.
Atabati et al. (2020) [42]	-	-	High intakes of acrylamide could marginal significantly reduce the risk of breast cancer.
Benisi-Kohansal et al. (2021) [76]	-	-	na
**Uterine Cancer**			
Hogervorst et al. (2007) [64]	327	21.0 ± 11.9 µg/day (0.32 ± 0.19 µg/kg bw/day)	No relationship was found between uterine cancer and dietary acrylamide exposure. However, increased acrylamide exposure in non-smokers women increased the risk of uterine cancer.
Larsson et al. (2009) [77]	687	24.6 ± 7.6 µg/day (0.38 ± 0.17 µg/kg bw/day)	na
Wilson et al. (2010) [69]	484	9–26 µg/day (0.13–0.42 µg/kg bw/day)	The risk of uterine cancer increased with increasing dietary acrylamide exposure.
Pelucchi et al. (2011) [71]	908	-	na
Obón-Santacana et al. (2014) [78]	1.382	23.7 ± 13.0 µg/day (0.4 ± 0.2 µg/kg bw/day)	No association was found between dietary acrylamide exposure and uterine cancer. However, increased exposure to acrylamide was associated with an increased risk of EC type I in both smokers and non-smokers.
Je (2015) [79]	2.099	-	No relationship was found between uterine cancer and dietary acrylamide exposure. However, among non-smokers, the risk of uterine cancer increased with high acrylamide exposure.
Pelucchi et al. (2015) [72]	2774	-	na
Hogervorst et al. (2016) [80]	393	21.3 ± 12.7 µg/day	No positive correlation was found between uterine cancer and dietary acrylamide exposure. However, a statistically significant relationship was found between exposure to acrylamide with a few nominal SNPs genes (CYP2E1: rs915906; rs2480258) and the deletions (GSTM1-GSTT1). This was explained as acrylamide exposure may contribute to the risk of developing uterine cancer.
Obón-Santacana et al. (2016) [81]	383	Hb-AA: 39.9 (pmol/g Hb) Hb-GA: 34.1 (pmol/g Hb)	na
Pelucchi et al. (2016) [82]	454	33.7 μg/day	na
Kotemori et al. (2018) [83]	161	7.1 ± 3.7 μg/day (0.14 ± 0.13 µg/kg bw/day)	na
Huang and Wang (2019) [84]	3228		The current meta-analysis did not support a significant association between acrylamide intake and endometrial cancer risk. However, women with high acrylamide exposure who never smoked had a higher risk of endometrial cancer.
Adani et al. (2020) [75]	-	-	While intermediate acrylamide exposure caused the highest risk of endometrial cancer, the relationship between exposure and cancer was linear and positive in never-smokers. High acrylamide exposure was associated with a linearly increased risk of endometrial cancer, particularly in never-smokers.
Benisi-Kohansal et al. (2021) [76]	-	-	na
**Ovarian Cancer**			
Pelucchi et al. (2006) [63]	1031	23.33 µg/day (0.37 µg/kg bw/day)	na
Hogervorst et al. (2007) [64]	300	21.0 ± 11.9 µg/day (0.32 ± 0.19 µg/kg bw/day)	A statistically significant association was found between dietary acrylamide exposure and ovarian cancer (especially in non-smokers).
Larsson et al. (2009) [85]	368	24.6 ± 7.6 µg/day (0.38 ± 0.17 µg/kg bw/day)	na
Wilson et al. (2010) [69]	416	9–26 µg/day (0.13–0.42 µg/kg bw/day)	No statistical relationship was found between dietary acrylamide exposure and ovarian cancer. However, it was stated that the risk of possible ovarian cancer may increase due to the risk of multiple tumors.
Pelucchi et al. (2011) [71]	908	-	na
Xie et al. (2013) [86]	263	Hb-AA: 112.6 pmol/g	na
Pelucchi et al. (2015) [72]	2010	-	na
Obón-Santacana et al. (2015) [87]	1191	21.3 µg/day (0.3 µg/kg bw/day)	na
Hogervorst et al. (2017) [88]	252	21.9 ± 13.1 µg/day	No statistically significant relationship was found between genetic variants and acrylamide exposure. However, a significant correlation was found between the nominal SNPs gene (HSD3B1/B2: rs4659175; rs10923823) and its proxies rs7546652, rs1047303 and rs6428830 and acrylamide exposure. It was stated that acrylamide may cause ovarian cancer due to its effect on sex hormones.
Kotemori et al. (2018) [83]	122	7.0 ± 3.7 μg/day (0.14 ± 0.13 µg/kg bw/day)	na
Adani et al. (2020) [75]	-	-	The risk of ovarian cancer slightly increased in individuals with dietary acrylamide exposure, particularly in never-smokers. High acrylamide intake caused a linear increase in the risk of ovarian and endometrial cancer, especially in never-smokers.
Benisi-Kohansal et al. (2021) [76]	-	-	na
Khodavandi, Alizadeh, and Razis (2021) [89]	-	-	na

na: no association; ER: Estrogen receptor; PR: Progesterone receptor; EC: Endometrial cancer; Hb-AA: Hemoglobin adducts of acrylamide, Hb-GA: Hemoglobin adducts of glycidamide.

**Table 2:** Epidemiological studies examining the effect of dietary acrylamide exposure on cancer types in the gastrointestinal system.

Type of Cancer—Reference	Cases	Mean Dietary Acrylamide Intake	Risk
**Esophageal Cancer**			
Pelucchi et al. (2006) [63]	395	27.47 µg/day (0.36 µg/kg bw/day)	na
Hogervorst et al. (2008) [91]	216	21.8 ± 12.1 µg/day (0.30 ± 0.18 µg/kg bw/day)	na
Lin, Lagergren, and Lu (2011) [92]	EC: 618 E-AC: 189 GEJ-AC:222 E-SCC: 167	E-AC: 37.6 ± 14.6 µg/day GEJ-AC: 37.5 ± 14.1 µg/day E-SCC: 38.5 ± 14.6 µg/day	All combined esophageal tumors increased with increasing acrylamide exposure. A statistically significant relationship was found especially in overweight and obese patients. A statistically significant relationship was found between E-SCC and dietary acrylamide exposure. This is much stronger in non-smokers with E-SCC.
Pelucchi et al. (2011) [71]	611	-	na
Luján-Barroso et al. (2014) [93]	EC: 341 E-AC: 142 E-SCC:176 Other: 23	26.22 ± 14.79 µg/day	Dietary acrylamide exposure increased the risk of E-AC cancer and tumors. However, since this increase was not linear, a statistically significant relationship could not be determined.
Pelucchi et al. (2015) [72]	1546	-	na
Liu et al. (2019) [94]	391	6.80 µg/day	na
Filippini et al. (2022) [95]	-	23 μg/day	na
**Gastric Cancer**			
Hogervorst et al. (2008) [91]	563	21.8 ± 12.1 µg/day (0.30 ± 0.18 µg/kg bw/day)	na
Hirvonen et al. (2010) [96]	224	36.8 μg/day	na
Pelucchi et al. (2016) [82]	787	-	na
Liu et al. (2019) [94]	2218	6.8 μg/day	na
Filippini et al. (2022) [95]	-	23 μg/day	na
**Colorectal Cancer**			
Mucci et al. (2003) [97]	Total:591 E: 346 K: 245	28.6 µg/day	An inverse relationship was found between dietary acrylamide exposure and colon cancer.
Mucci, Adami, and Wolk (2006) [90]	Total: 741 Kolon: 504 Rektum: 237	24.6 µg/day (0.38 µg/kg bw/day)	na
Pelucchi et al. (2006) [63]	Total: 2280 Kolon:1394 Rektum:886	27.69 µg/day (0.40 µg/kg bw/day)	na
Hogervorst et al. (2008) [91]	2190	21.8 ± 12.1 µg/day (0.30 ± 0.18 µg/kg bw/day)	na
Larsson et al. (2009) [98]	Kolon: 410 Rektum: 266	36.1 ± 9.6 μg/day	na
Hirvonen et al. (2010) [96]	316	21.9–55.7 μg/day	na
Pelucchi et al. (2011) [71]	Kolorektum: 5887 Kolon: 3813 Rektum: 1899	-	na
Hogervorst et al. (2007) [64]	M: 341 W: 282	E: 23 μg/day (0.29 µg/kg bw/day) K: 20 μg/day (0.29 µg/kg bw/day)	No statistically significant relationship was found between dietary acrylamide exposure and total colorectal cancer. There was also an inverse relationship between acrylamide exposure and tumors with the APC mutation among women.
Pelucchi et al. (2015) [72]	6794	-	na
Liu et al. (2019) [94]	2470	6.8 μg/day	na
Filippini et al. (2022) [95]	-	23 μg/day	na
**Pancreatic Cancer**			
Hogervorst et al. (2008) [91]	349	21.8 ± 12.1 µg/day (0.30 ± 0.18 µg/kg bw/day)	na
Hirvonen et al. (2010) [96]	192	21.9–55.7 μg/day	na
Pelucchi et al. (2011) [99]	326	33.51 ± 17.42 µg/day	na
Obón-Santacana et al. (2013) [100]	865	26.22 µg/day 0.38 µg/kg bw/day	na
Pelucchi et al. (2015) [72]	1732	-	na
Pelucchi et al. (2017) [101]	1975	-	na
Kito et al. (2020) [102]	576	6.90 μg/day	na
Filippini et al. (2022) [95]	-	23 μg/day	na
**Liver Cancer**			
Zha et al. (2020) [103]	744	6.90 μg/day	na
Filippini et al. (2022) [95]	-	23 μg/day	na
**Digestive System Cancers**			
Liu et al. (2017) [104]	131	14.6 ± 8.2 μg/day	The relationship between dietary acrylamide exposure and digestive system cancers was found to be statistically significant.

na: No association; EC: Esophagus cancer; E-AC: Adenocarcinoma of the esophagus. E-SCC: Squamous cell carcinoma of the esophagus. GEJ-AC: Adenocarcinoma of gastroesophageal junctional; M: Man; W: Woman.

**Table 3:** Epidemiological studies examining the effect of dietary acrylamide exposure on urinary system cancer types.

Type of Cancer—Reference	Cases	Mean Dietary Acrylamide Intake	Risk
**Prostate Cancer**			
Pelucchi et al. (2006) [63]	1294	25.59 µg/day (0.33 µg/kg bw/day)	na
Hogervorst et al. (2008) [106]	2246	22.4 µg/day (0.29 µg/kg bw/day)	na
Wilson et al. (2009) [107]	1489	43.8 ± 13.7 µg/day (0.54 ± 0.18 µg/kg bw/day) Hb-AA: 54.7 pmol/g globin	na
Larsson et al. (2009) [105]	2696	36.1 ± 9.6 μg/day	na
Hirvonen et al. (2010) [96]	799	21.9–55.7 μg/day	na
Pelucchi et al. (2011) [71]	7735	-	na
Wilson et al. (2012) [108]	5025	10.5–40.1 μg/day	na
Pelucchi et al. (2015) [72]	13,559	-	na
Perloy et al. (2018) [109]	948	22.9 μg/day	na
Ikeda et al. (2021) [110]	1195	6.40 μg/day	na
Filippini et al. (2022) [95]	-	23 μg/day	na
**Bladder Cancer**			
Mucci et al. (2003) [97]	233	29.4 ± 0.9 μg/day	na
Hogervorst et al. (2008) [106]	1210	9.5–40.8 μg/day	na
Hirvonen et al. (2010) [96]	365	21.9–55.7 μg/day	na
Pelucchi et al. (2011) [71]	1473	-	na
Pelucchi et al. (2015) [72]	1838	-	na
Ikeda et al. (2021) [110]	392	6.40 μg/day	na
Filippini et al. (2022) [95]	-	23 μg/day	na
**Renal Cancer**			
Mucci et al. (2003) [97]	133	28.4 ± 1.2 μg/day	na
Mucci et al. (2004) [111]	379	27.6 ± 0.7 μg/day	na
Pelucchi et al. (2007) [112]	767	37 μg/day (0.475 µg/kg bw/day)	na
Hogervorst et al. (2008) [106]	339	9.5–40.8 μg/day	A positive correlation was found between dietary acrylamide exposure and renal cancer.
Hirvonen et al. (2010) [96]	184	21.9–55.7 μg/day	na
Pelucchi et al. (2011) [71]	1618	-	na
Pelucchi et al. (2015) [72]	1802	-	na
Graff et al. (2018) [113]	629	Female: 15.8 μg/day Male: 21.7 μg/day	na
McCullough et al. (2019) [114]	412	22.6 μg/day	na
Jiang et al. (2020) [115]	2843	-	na
Ikeda et al. (2021) [110]	208	6.40 μg/day (0.12 µg/kg bw/day)	na
Filippini et al. (2022) [95]	-	23 μg/day	na

na: no association.

**Table 4:** Studies examining the effect of dietary acrylamide exposure on respiratory system cancer types.

Type of Cancer—Reference	Cases	Mean Dietary Acrylamide Intake	Risk
**Oral cavity/pharyngeal cancer**			
Pelucchi et al. (2006) [63]	749	29.24 μg/day (0.40 µg/kg bw/day)	na
Schouten et al. (2009)	OCC: 101 (M: 61-W:40) OHC: 83 (M: 41-W:22)	OCC-M: 18.8 μg/day (0.25 µg/kg bw/day) OCC-W: 18.8 μg/day (0.28 µg/kg bw/day) OHC-M: 18.3 μg/day (0.23 µg/kg bw/day) OHC-W: 20.7 μg/day (0.32 µg/kg bw/day)	No statistically significant relationship was found between dietary acrylamide exposure and OCC cancer. However, it was stated that acrylamide exposure may increase the risk of OCC cancer in non-smokers. OHC: na
Pelucchi et al. (2015) [72]	933	-	na
Filippini et al. (2022) [95]	-	23 μg/day	na
**Throat Cancer**			
Pelucchi et al. (2006) [63]	527	27.10 μg/day (0.36 µg/kg bw/day)	na
Schouten et al. (2009) [116]	LC: 180 (M: 170-W:10)	LC-M: 23.3 μg/day (0.31 µg/kg bw/day) LC-W: 21.0 μg/day (0.35 µg/kg bw/day)	na
Pelucchi et al. (2015) [72]	707	-	na
Filippini et al. (2022) [95]	-	23 μg/day	na
**Lung Cancer**			
Hogervorst et al. (2009) [117]	M: 1.600 W: 295	-	M: na W: A very strong inverse relationship was found between dietary acrylamide exposure and adenocarcinoma.
Hirvonen et al. (2010) [96]	1.703	21.9–55.7 μg/day	A positive correlation was found between dietary acrylamide exposure and lung cancer.
Pelucchi et al. (2015) [72]	3.598	-	na
Liu et al. (2020) [118]	M: 1187 W: 485	Female: 6.8 μg/day Male: 7.0 μg/day	na
Filippini et al. (2022) [95]	-	23 μg/day	na
**Respiratory System Cancers**			
Liu et al. (2017) [104]	104	14.6 ± 8.2 μg/day	The increase in the number of respiratory system cancer cases with dietary acrylamide exposure was found to be statistically significant.

na: No association; OCC: Oral cavity carnicoma; OHC: Oropharynx-hypopharynx cancer; LC: Larynx cancer; M: Man; W: Woman.

**Table 5:** Studies examining the impact of dietary acrylamide exposure on cancer types in other systems.

Type of Cancer—Reference	Cases	Mean Dietary Acrylamide Intake	Risk
**Brain Cancer**			
Hogervorst et al. (2009) [119]	216	22.1 μg/day (0.30 µg/kg bw/day)	na
Filippini et al. (2022) [95]	-	23 μg/day	na
**Thyroid Cancer**			
Schouten et al. (2009) [116]	M: 19 W: 47	M: 22.1 μg/day (0.28 µg/kg bw/day) W: 21.6 μg/day (0.33 µg/kg bw/day)	na
Filippini et al. (2022) [95]	-	23 μg/day	na
**Lymphatic Malignancies**			
Hirvonen et al. (2010) [96]	L: 175	21.9–55.7 μg/day	na
Bongers et al. (2012) [120]	Total LM: 1233 CLL:200 DLCL:259 MM:323 FL:89 WMI:89 MCL-M:56 T-cell-M:54	CLL: 20.0–21.0 μg/day DLCL: 21.0–23.0 μg/day MM: 21.0–25.0 μg/day FL: 23.0–26.0 μg/day WMI: 23.0–26.0 μg/day MCL-M: 20.0–22.0 μg/day T-cell-M: 22.0–27.0 μg/day	A statistically significant relationship was only found between dietary acrylamide exposure and MM-M.
Pelucchi et al. (2015) [72]	1208	-	na
Zha et al. (2021) [121]	ML: 326 MM: 126 L: 224	6.90 μg/day	na
Filippini et al. (2022) [95]	-	23 μg/day	na
**All Types of Cancer**			
Liu et al. (2017) [104]	131	14.6 ± 8.2 μg/day	The relationship between dietary acrylamide exposure and all cancer types was found to be statistically significant.
**Skin Cancer**			
Lipunova et al. (2017) [122]	CMM-M:241 SSM-M:90 NM-M:40 CMM-W:236 SSM-W:102 NM-W:30	23.9 μg/day 24.1 μg/day 27.5 μg/day 21.2 μg/day 20.5 μg/day 21.5 μg/day	Modeling with an increase of 10 μg/day acrylamide increased the risk of CMM-M among men. But this increase was not linear. A weaker correlation was found among non-smokers in terms of CMM-M. The increased risk of NM-M among men was statistically significant. Among non-smokers, this relationship was much stronger. No significant relationship was found between SSM-M and acrylamide exposure among men. According to the same modeling, no statistically significant data could be obtained between acrylamide exposure and melanoma risk in women.

na: No association, LM: Lymphatic Malignancies; ML: Malignant lymphoma CLL: Chronic lymphocytic leukemia; DLCL: Diffuse large cell lymphoma; WMI: Waldenstrom macroglobulinemia and immunocytoma; FL: Follicular lymphoma; MCL: Mantle cell lymphoma; T-cell: T-cell lymphomas; MM: Multiple myeloma, M: Melanoma, CMM: Cutaneous malignant melanoma, SSM: Superficial spreading melanoma, NM: Nodular melanoma, L: Leukemia, M: Man; W: Woman.

## Data Availability

Not applicable.
